# Diaqua-1κ*O*,3κ*O*-di-μ-cyanido-1:2κ^2^ *N*:*C*;2:3κ^2^ *C*:*N*-dicyanido-2κ^2^ *N*-bis­{4,4′-dibromo-2,2′-[propane-1,2-diylbis(nitrilo­methyl­idyne)]diphenolato}-1κ^4^ *O*,*N*,*N*′,*O*′;3κ^4^ *O*,*N*,*N*′,*O*′-1,3-dimanganese(III)-2-nickel(II)

**DOI:** 10.1107/S1600536808012749

**Published:** 2008-05-07

**Authors:** Zhen-Hai Sun, Gui-Bin Yang, Ling-Bo Meng, Shen Chen

**Affiliations:** a School of Chemistry and Life Sciences, Harbin University, Harbin 150080, People’s Republic of China

## Abstract

In the title compound, [Mn_2_Ni(C_17_H_14_Br_2_N_2_O_2_)_2_(CN)_4_(H_2_O)_2_] or [{Mn(C_17_H_14_Br_2_N_2_O_2_)(H_2_O)}_2_(μ-CN)_2_{Ni(CN)_2_}], each Mn^III^ atom is chelated by a Schiff base ligand *via* two N and two O atoms and is additionally coordinated by a water mol­ecule to give a slightly distorted octa­hedral geometry. Two such Mn^III^ ions are linked by a square-planar Ni(CN)_4_ unit, which lies on an inversion centre. A two-dimensional network is formed by O—H⋯O and O—H⋯N hydrogen bonds.

## Related literature

For related literature, see: Garnovskii *et al.* (1993 ▶); Huang *et al.* (2002 ▶); Bhadbhade & Srinivas (1993 ▶); Bunce *et al.* (1998 ▶).
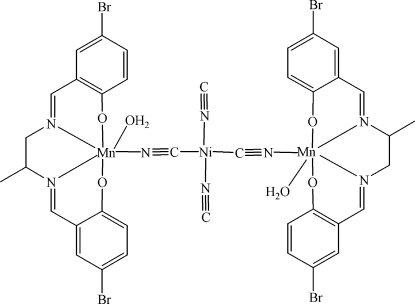


## Experimental

### 

#### Crystal data

[Mn_2_Ni(C_17_H_14_Br_2_N_2_O_2_)_2_(CN)_4_(H_2_O)_2_]
*M* *_r_* = 1184.95
Monoclinic, 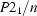

*a* = 11.619 (2) Å
*b* = 13.514 (3) Å
*c* = 14.741 (3) Å
β = 112.04 (3)°
*V* = 2145.5 (7) Å^3^
*Z* = 2
Mo *K*α radiation
μ = 4.79 mm^−1^
*T* = 293 (2) K
0.12 × 0.10 × 0.08 mm

#### Data collection

Bruker APEXII CCD area-detector diffractometer
Absorption correction: multi-scan (*SADABS*; Bruker, 2001 ▶) *T*_min_ = 0.597, *T*_max_ = 0.700
13467 measured reflections
3712 independent reflections
2268 reflections with *I* > 2σ(*I*)
*R*_int_ = 0.085

#### Refinement

*R*[*F* ^2^ > 2σ(*F* ^2^)] = 0.065
*wR*(*F* ^2^) = 0.175
*S* = 1.00
3712 reflections
277 parameters
3 restraints
H atoms treated by a mixture of independent and constrained refinement
Δρ_max_ = 0.81 e Å^−3^
Δρ_min_ = −0.69 e Å^−3^

### 

Data collection: *APEX2* (Bruker, 2004 ▶); cell refinement: *SAINT-Plus* (Bruker, 2001 ▶); data reduction: *SAINT-Plus*; program(s) used to solve structure: *SHELXS97* (Sheldrick, 2008 ▶); program(s) used to refine structure: *SHELXL97* (Sheldrick, 2008 ▶); molecular graphics: *SHELXTL* (Sheldrick, 2008 ▶); software used to prepare material for publication: *SHELXTL*.

## Supplementary Material

Crystal structure: contains datablocks I, global. DOI: 10.1107/S1600536808012749/cf2192sup1.cif

Structure factors: contains datablocks I. DOI: 10.1107/S1600536808012749/cf2192Isup2.hkl

Additional supplementary materials:  crystallographic information; 3D view; checkCIF report

## Figures and Tables

**Table 1:** Hydrogen-bond geometry (Å, °)

*D*—H⋯*A*	*D*—H	H⋯*A*	*D*⋯*A*	*D*—H⋯*A*
O3—H1*W*⋯O1^i^	0.82 (7)	2.06 (7)	2.860 (7)	165 (10)
O3—H2*W*⋯N2^ii^	0.82 (4)	2.00 (2)	2.803 (8)	167 (8)

Symmetry codes: (i) 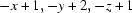; (ii) 
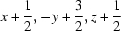
.

**Fig. 1.**
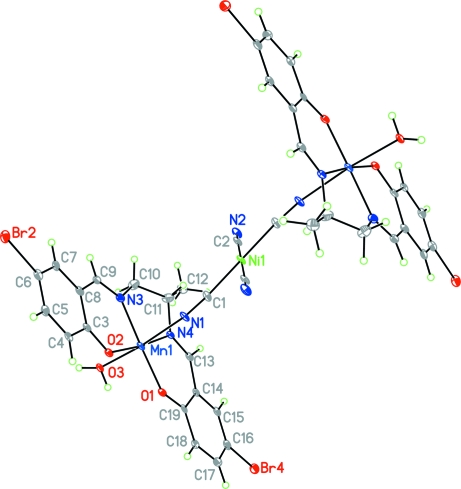
The molecular structure of (I), drawn with 30% probability displacement ellipsoids for the non-hydrogen atoms. [Symmetry code for unlabelled atoms: -x, 2 - y, -z.]

**Fig. 2.**
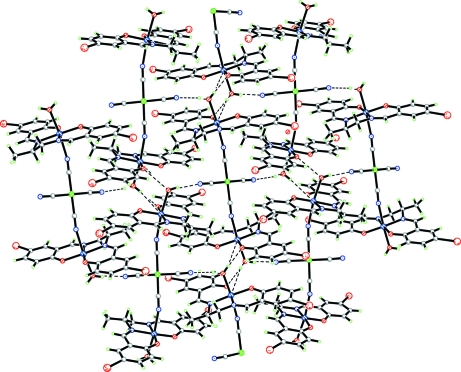
Two-dimensional network formed by hydrogen bonds (dashed lines).

## supplementary crystallographic information

### Comment

Schiff bases as ligands have been studied for a long time due to their easy synthesis and versatile complexing abilities. They play an important role in the development of coordination chemistry as well as inorganic biochemistry, catalysis, optical materials and so on (Garnovskii *et al.*, 1993; Huang *et al.*, 2002). Considerable attention has been focused on the syntheses and structures of manganese(III) complexes. Manganese complexes with multidentate Schiff base ligands have aroused particular interest because this metal can exhibit several oxidation states and may provide the basis of models for active sites of biological systems. On the other hand, the main attention in optically active Schiff base complexes is concentrated on their catalytic abilities in stereoselective synthesis (Bhadbhade & Srinivas, 1993; Bunce *et al.*, 1998). In this paper, we report the structure of the title compound, (I).

As shown in Fig. 1, each Mn^III^ atom is chelated by a Schiff base ligand *via* two N and two O atoms and is additionally coordinated by a water molecule to give a slightly distorted octahedral geometry, in which the Schiff base lies in the equatorial plane. Two such Mn^III^ ions are linked by a square-planar Ni(CN)_4_ unit, which lies on an inversion centre. The cyanido and aqua ligands lie in the axial coordination sites. The Mn—N and Mn—O axial bond lengths are much longer than the equatorial ones. A two-dimensional network is formed by O—H···O and O—H···N hydrogen bonds, as shown in Fig. 2.

### Experimental

A mixture of manganese(III) acetate (1 mmol), *N*,*N*'-bis(2-hydroxy-5-bromobenzyl)-1,2-diaminopropane (1 mmol) and dipotassium tetracyanidonickelate(II) (1 mmol) in 20 ml me thanol was refluxed for two hours. The cooled solution was filtered and the filtrate was allowed to evaporate naturally at room temperature. Two day later, brown blocks of (I) were obtained with a yield of 16%. Anal. Calc. for C_38_H_32_Br_4_Mn_2_N_8_NiO_6_: C 38.48, H 2.70, N 9.45%; Found: C 38.42, H 2.64, N 9.38.

### Refinement

All C-bound H atoms were placed in calculated positions with C—H = 0.93 Å and refined as riding with *U*_iso_(H) = 1.2*U*_eq_(C). H atoms of H_2_O were located in a difference density map and were refined with a distance restraint O—H = 0.82 (1) Å and with *U*_iso_(H) = 0.08 Å^2^.

### Crystal data

**Table:**

[Mn_2_Ni(C_17_H_14_Br_2_N_2_O_2_)_2_(CN)_4_(H_2_O)_2_]	*F*_000_ = 1164
*M**_r_* = 1184.95	*D*_x_ = 1.834 Mg m^−^^3^
Monoclinic, *P*2_1_/*n*	Mo *K*α radiation λ = 0.71073 Å
Hall symbol: -P 2yn	Cell parameters from 3712 reflections
*a* = 11.619 (2) Å	θ = 3.0–25.1º
*b* = 13.514 (3) Å	µ = 4.79 mm^−^^1^
*c* = 14.741 (3) Å	*T* = 293 (2) K
β = 112.04 (3)º	Block, brown
*V* = 2145.5 (7) Å^3^	0.12 × 0.10 × 0.08 mm
*Z* = 2	

### Data collection

**Table:**

Bruker APEXII CCD area-detector diffractometer	3712 independent reflections
Radiation source: fine-focus sealed tube	2268 reflections with *I* > 2σ(*I*)
Monochromator: graphite	*R*_int_ = 0.085
*T* = 293(2) K	θ_max_ = 25.1º
φ and ω scans	θ_min_ = 3.0º
Absorption correction: multi-scan(SADABS; Bruker, 2001)	*h* = −13→12
*T*_min_ = 0.597, *T*_max_ = 0.700	*k* = −16→15
13467 measured reflections	*l* = −17→17

### Refinement

**Table:**

Refinement on *F*^2^	Secondary atom site location: difference Fourier map
Least-squares matrix: full	Hydrogen site location: inferred from neighbouring sites
*R*[*F*^2^ > 2σ(*F*^2^)] = 0.065	H atoms treated by a mixture of independent and constrained refinement
*wR*(*F*^2^) = 0.175	*w* = 1/[σ^2^(*F*_o_^2^) + (0.085*P*)^2^] where *P* = (*F*_o_^2^ + 2*F*_c_^2^)/3
*S* = 1.00	(Δ/σ)_max_ < 0.001
3712 reflections	Δρ_max_ = 0.81 e Å^−^^3^
277 parameters	Δρ_min_ = −0.69 e Å^−^^3^
3 restraints	Extinction correction: none
Primary atom site location: structure-invariant direct methods	

### Special details

**Table:**

Geometry. All e.s.d.'s (except the e.s.d. in the dihedral angle between two l.s. planes) are estimated using the full covariance matrix. The cell e.s.d.'s are taken into account individually in the estimation of e.s.d.'s in distances, angles and torsion angles; correlations between e.s.d.'s in cell parameters are only used when they are defined by crystal symmetry. An approximate (isotropic) treatment of cell e.s.d.'s is used for estimating e.s.d.'s involving l.s. planes.
Refinement. Refinement of *F*^2^ against ALL reflections. The weighted *R*-factor *wR* and goodness of fit *S* are based on *F*^2^, conventional *R*-factors *R* are based on *F*, with *F* set to zero for negative *F*^2^. The threshold expression of *F*^2^ > σ(*F*^2^) is used only for calculating *R*-factors(gt) *etc*. and is not relevant to the choice of reflections for refinement. *R*-factors based on *F*^2^ are statistically about twice as large as those based on *F*, and *R*- factors based on ALL data will be even larger.

### Fractional atomic coordinates and isotropic or equivalent isotropic displacement parameters (Å^2^)

**Table:**

	*x*	*y*	*z*	*U*_iso_*/*U*_eq_	
Ni1	0.0000	1.0000	0.0000	0.0349 (4)	
Mn1	0.29546 (11)	0.95323 (8)	0.36729 (8)	0.0306 (4)	
Br2	−0.07326 (10)	1.37224 (7)	0.43825 (7)	0.0583 (4)	
Br4	0.75942 (10)	0.61247 (7)	0.30713 (8)	0.0636 (4)	
C1	0.1213 (8)	0.9936 (5)	0.1259 (5)	0.036 (2)	
C2	−0.0637 (8)	0.8806 (6)	0.0275 (6)	0.039 (2)	
C3	0.2230 (8)	1.1458 (5)	0.4133 (5)	0.035 (2)	
C4	0.2475 (8)	1.2492 (5)	0.4247 (5)	0.0338 (19)	
H4	0.3241	1.2728	0.4279	0.041*	
C5	0.1609 (9)	1.3147 (6)	0.4311 (6)	0.043 (2)	
H5	0.1781	1.3821	0.4359	0.052*	
C6	0.0470 (9)	1.2810 (6)	0.4305 (6)	0.043 (2)	
C7	0.0187 (9)	1.1818 (6)	0.4197 (6)	0.046 (2)	
H7	−0.0578	1.1601	0.4184	0.056*	
C8	0.1033 (8)	1.1135 (5)	0.4107 (6)	0.038 (2)	
C9	0.0682 (8)	1.0107 (6)	0.3967 (6)	0.037 (2)	
H9	−0.0076	0.9939	0.4003	0.045*	
C10	0.0874 (10)	0.8352 (6)	0.3699 (9)	0.070 (3)	
H10A	0.1148	0.8044	0.4340	0.084*	
H10B	−0.0026	0.8335	0.3419	0.084*	
C11	0.1358 (9)	0.7815 (6)	0.3084 (9)	0.069 (3)	
H11	0.0896	0.8087	0.2432	0.082*	
C12	0.1046 (9)	0.6736 (6)	0.2966 (8)	0.063 (3)	
H12A	0.1564	0.6386	0.3541	0.094*	
H12B	0.1185	0.6485	0.2406	0.094*	
H12C	0.0190	0.6645	0.2874	0.094*	
C13	0.3444 (7)	0.7543 (5)	0.3197 (5)	0.034 (2)	
H13	0.3200	0.6888	0.3050	0.041*	
C14	0.4691 (7)	0.7788 (5)	0.3300 (5)	0.035 (2)	
C15	0.5439 (8)	0.7030 (6)	0.3193 (5)	0.039 (2)	
H15	0.5142	0.6384	0.3117	0.047*	
C16	0.6589 (9)	0.7208 (7)	0.3196 (6)	0.050 (3)	
C17	0.7050 (9)	0.8158 (7)	0.3290 (6)	0.050 (2)	
H17	0.7826	0.8279	0.3265	0.060*	
C18	0.6340 (8)	0.8932 (6)	0.3422 (6)	0.041 (2)	
H18	0.6660	0.9571	0.3508	0.049*	
C19	0.5152 (8)	0.8769 (6)	0.3428 (5)	0.034 (2)	
N1	0.1906 (6)	0.9902 (4)	0.2061 (4)	0.0384 (18)	
N2	−0.0940 (7)	0.8037 (5)	0.0442 (5)	0.048 (2)	
N3	0.1310 (6)	0.9397 (5)	0.3797 (5)	0.0411 (18)	
N4	0.2646 (6)	0.8130 (4)	0.3287 (4)	0.0316 (16)	
O1	0.4523 (5)	0.9530 (3)	0.3561 (3)	0.0328 (13)	
O2	0.3094 (5)	1.0873 (3)	0.4049 (4)	0.0327 (13)	
O3	0.3787 (5)	0.9022 (4)	0.5249 (4)	0.0368 (14)	
H1W	0.434 (5)	0.942 (4)	0.552 (7)	0.080*	
H2W	0.398 (7)	0.8437 (15)	0.533 (7)	0.080*	

### Atomic displacement parameters (Å^2^)

**Table:**

	*U*^11^	*U*^22^	*U*^33^	*U*^12^	*U*^13^	*U*^23^
Ni1	0.0383 (9)	0.0266 (8)	0.0214 (7)	−0.0002 (6)	−0.0099 (6)	0.0005 (6)
Mn1	0.0332 (7)	0.0231 (6)	0.0221 (6)	0.0005 (5)	−0.0051 (5)	−0.0009 (5)
Br2	0.0739 (8)	0.0485 (6)	0.0467 (6)	0.0242 (5)	0.0159 (5)	−0.0025 (4)
Br4	0.0553 (7)	0.0701 (7)	0.0566 (7)	0.0211 (5)	0.0109 (5)	−0.0140 (5)
C1	0.055 (6)	0.013 (4)	0.028 (4)	−0.001 (4)	0.003 (4)	0.000 (3)
C2	0.039 (5)	0.035 (5)	0.024 (4)	0.003 (4)	−0.010 (4)	0.000 (4)
C3	0.037 (5)	0.029 (4)	0.023 (4)	0.006 (4)	−0.006 (4)	−0.003 (3)
C4	0.041 (5)	0.032 (4)	0.020 (4)	−0.008 (4)	0.001 (4)	0.001 (3)
C5	0.060 (6)	0.030 (5)	0.032 (5)	0.010 (4)	0.009 (4)	−0.004 (4)
C6	0.059 (6)	0.026 (4)	0.033 (5)	0.006 (4)	0.004 (4)	0.001 (4)
C7	0.053 (6)	0.057 (6)	0.023 (4)	0.008 (5)	0.006 (4)	−0.007 (4)
C8	0.044 (5)	0.031 (5)	0.025 (4)	0.009 (4)	−0.003 (4)	0.000 (3)
C9	0.035 (5)	0.037 (5)	0.033 (4)	0.000 (4)	0.006 (4)	−0.003 (4)
C10	0.069 (8)	0.043 (6)	0.112 (10)	−0.017 (5)	0.049 (7)	−0.028 (6)
C11	0.047 (7)	0.040 (6)	0.117 (10)	−0.004 (5)	0.030 (6)	−0.027 (6)
C12	0.063 (7)	0.041 (5)	0.077 (7)	−0.007 (5)	0.019 (6)	−0.010 (5)
C13	0.042 (5)	0.019 (4)	0.029 (4)	0.001 (4)	0.001 (4)	−0.002 (3)
C14	0.035 (5)	0.035 (5)	0.022 (4)	0.008 (4)	−0.002 (4)	−0.007 (3)
C15	0.047 (6)	0.038 (5)	0.020 (4)	0.003 (4)	0.000 (4)	0.001 (3)
C16	0.053 (6)	0.053 (6)	0.028 (5)	0.020 (5)	−0.003 (4)	−0.008 (4)
C17	0.045 (6)	0.055 (6)	0.045 (6)	−0.001 (5)	0.012 (5)	−0.010 (5)
C18	0.044 (6)	0.046 (5)	0.026 (4)	−0.002 (4)	0.006 (4)	−0.004 (4)
C19	0.037 (5)	0.042 (5)	0.011 (4)	0.009 (4)	−0.005 (3)	−0.003 (3)
N1	0.045 (4)	0.027 (3)	0.025 (4)	−0.006 (3)	−0.009 (3)	0.000 (3)
N2	0.058 (5)	0.030 (4)	0.039 (4)	−0.010 (4)	0.000 (4)	−0.004 (3)
N3	0.038 (4)	0.033 (4)	0.045 (4)	−0.002 (3)	0.007 (3)	−0.010 (3)
N4	0.030 (4)	0.028 (4)	0.027 (4)	0.000 (3)	0.000 (3)	−0.001 (3)
O1	0.032 (3)	0.030 (3)	0.026 (3)	0.003 (2)	−0.001 (2)	0.001 (2)
O2	0.034 (3)	0.029 (3)	0.027 (3)	0.001 (2)	0.002 (2)	0.001 (2)
O3	0.042 (4)	0.029 (3)	0.025 (3)	−0.003 (3)	−0.004 (3)	−0.003 (2)

### Geometric parameters (Å, °)

**Table:**

Ni1—C1	1.865 (8)	C9—H9	0.930
Ni1—C1^i^	1.865 (8)	C10—C11	1.431 (13)
Ni1—C2^i^	1.882 (9)	C10—N3	1.489 (10)
Ni1—C2	1.882 (9)	C10—H10A	0.970
Mn1—O2	1.884 (5)	C10—H10B	0.970
Mn1—O1	1.890 (6)	C11—N4	1.475 (11)
Mn1—N4	1.973 (6)	C11—C12	1.497 (11)
Mn1—N3	1.995 (7)	C11—H11	0.980
Mn1—O3	2.264 (5)	C12—H12A	0.960
Mn1—N1	2.282 (6)	C12—H12B	0.960
Br2—C6	1.899 (9)	C12—H12C	0.960
Br4—C16	1.925 (9)	C13—N4	1.265 (9)
C1—N1	1.154 (9)	C13—C14	1.438 (11)
C2—N2	1.153 (9)	C13—H13	0.930
C3—O2	1.319 (9)	C14—C15	1.389 (11)
C3—C4	1.423 (10)	C14—C19	1.415 (10)
C3—C8	1.443 (12)	C15—C16	1.356 (12)
C4—C5	1.370 (11)	C15—H15	0.930
C4—H4	0.930	C16—C17	1.378 (12)
C5—C6	1.397 (12)	C17—C18	1.391 (12)
C5—H5	0.930	C17—H17	0.930
C6—C7	1.375 (11)	C18—C19	1.401 (12)
C7—C8	1.390 (11)	C18—H18	0.930
C7—H7	0.930	C19—O1	1.319 (9)
C8—C9	1.442 (10)	O3—H1W	0.82 (7)
C9—N3	1.286 (10)	O3—H2W	0.82 (4)
			
C1—Ni1—C1^i^	180	N3—C10—H10B	109.7
C1—Ni1—C2^i^	92.5 (3)	H10A—C10—H10B	108.2
C1^i^—Ni1—C2^i^	87.5 (3)	C10—C11—N4	109.6 (8)
C1—Ni1—C2	87.5 (3)	C10—C11—C12	115.6 (9)
C1^i^—Ni1—C2	92.5 (3)	N4—C11—C12	119.2 (8)
C2^i^—Ni1—C2	180	C10—C11—H11	103.4
O2—Mn1—O1	92.8 (2)	N4—C11—H11	103.4
O2—Mn1—N4	174.4 (3)	C12—C11—H11	103.4
O1—Mn1—N4	92.9 (2)	C11—C12—H12A	109.5
O2—Mn1—N3	92.3 (2)	C11—C12—H12B	109.5
O1—Mn1—N3	174.6 (2)	H12A—C12—H12B	109.5
N4—Mn1—N3	82.0 (3)	C11—C12—H12C	109.5
O2—Mn1—O3	92.0 (2)	H12A—C12—H12C	109.5
O1—Mn1—O3	92.0 (2)	H12B—C12—H12C	109.5
N4—Mn1—O3	88.0 (2)	N4—C13—C14	126.4 (7)
N3—Mn1—O3	86.2 (3)	N4—C13—H13	116.8
O2—Mn1—N1	92.8 (2)	C14—C13—H13	116.8
O1—Mn1—N1	93.7 (2)	C15—C14—C19	119.0 (8)
N4—Mn1—N1	86.7 (2)	C15—C14—C13	117.8 (7)
N3—Mn1—N1	87.7 (3)	C19—C14—C13	123.1 (7)
O3—Mn1—N1	172.4 (2)	C16—C15—C14	121.7 (8)
N1—C1—Ni1	175.8 (8)	C16—C15—H15	119.1
N2—C2—Ni1	174.3 (8)	C14—C15—H15	119.1
O2—C3—C4	118.2 (7)	C15—C16—C17	120.8 (9)
O2—C3—C8	125.2 (7)	C15—C16—Br4	119.8 (7)
C4—C3—C8	116.6 (7)	C17—C16—Br4	119.4 (8)
C5—C4—C3	121.5 (8)	C16—C17—C18	119.0 (9)
C5—C4—H4	119.2	C16—C17—H17	120.5
C3—C4—H4	119.2	C18—C17—H17	120.5
C4—C5—C6	120.5 (7)	C17—C18—C19	121.4 (8)
C4—C5—H5	119.8	C17—C18—H18	119.3
C6—C5—H5	119.8	C19—C18—H18	119.3
C7—C6—C5	120.2 (8)	O1—C19—C18	118.7 (7)
C7—C6—Br2	119.4 (7)	O1—C19—C14	123.2 (8)
C5—C6—Br2	120.3 (6)	C18—C19—C14	118.1 (7)
C6—C7—C8	120.8 (9)	C1—N1—Mn1	165.7 (7)
C6—C7—H7	119.6	C9—N3—C10	121.9 (8)
C8—C7—H7	119.6	C9—N3—Mn1	125.6 (6)
C7—C8—C9	118.8 (8)	C10—N3—Mn1	112.4 (5)
C7—C8—C3	120.4 (7)	C13—N4—C11	121.7 (6)
C9—C8—C3	120.8 (7)	C13—N4—Mn1	124.9 (6)
N3—C9—C8	126.6 (8)	C11—N4—Mn1	113.3 (5)
N3—C9—H9	116.7	C19—O1—Mn1	128.4 (5)
C8—C9—H9	116.7	C3—O2—Mn1	128.3 (5)
C11—C10—N3	109.9 (8)	Mn1—O3—H1W	105 (7)
C11—C10—H10A	109.7	Mn1—O3—H2W	116 (7)
N3—C10—H10A	109.7	H1W—O3—H2W	116 (8)
C11—C10—H10B	109.7		

Symmetry codes: (i) −*x*, −*y*+2, −*z*.

### Hydrogen-bond geometry (Å, °)

**Table:**

*D*—H···*A*	*D*—H	H···*A*	*D*···*A*	*D*—H···*A*
O3—H1W···O1^ii^	0.82 (7)	2.06 (7)	2.860 (7)	165 (10)
O3—H2W···N2^iii^	0.82 (4)	2.00 (2)	2.803 (8)	167 (8)

Symmetry codes: (ii) −*x*+1, −*y*+2, −*z*+1; (iii) *x*+1/2, −*y*+3/2, *z*+1/2.
